# Supplementary material for: “Omics” and Postmortem Interval Estimation: A Systematic Review
Source: Int J Mol Sci. 2025 Jan 25;26(3):1034. doi: 10.3390/ijms26031034 (PMC11817326; doi:10.3390/ijms26031034)
Supplement: Supplementary file 1 [file ijms-26-01034-s001.zip › Sup_Material_Up_Down_regulation_PMI - Table S3.pdf]

		Short PMI		Intermediate PMI		Long PMI	
**Down-regulation pattern**	↓	3-o-metylguanosine,<br>ACY1,<br>Adenosine kinase,<br>Adenosine triphosphate synthase,<br>Alpha-enolase,<br>AKI1,<br>Amine oxidase,<br>ATP2A2 (SERCA2),<br>ATP5MF,<br>ATP5MG,<br>ATP5PB,<br>BAG3,<br>Basic proline-rich protein-like,<br>Cadaverine,<br>Cardiac myosin heavy chain 5,<br>Citric acid,<br>Desmin,<br>DHRS7C,<br>eEF1A2,<br>eEF2,<br>Enolase,<br>GAPDH,<br>Glutathione synthetase,<br>GPS1,<br>Guanosine 5-monophosphate,<br>Inosine 5-monophosphate,<br>Interferon omega 5 precursor,	Isomaltose,<br>IPO5,<br>LOC102151723,<br>LOC100156325,<br>MURC,<br>MYOZ1,<br>MYOZ3,<br>Nicotinamide,<br>Olfactory receptor 2G3-like,<br>PDLIM5<br>PDLIM7,<br>PDMI3,<br>PRDX2,<br>Pyruvate,<br>RAB10,<br>Rho GTPase-activating protein 24,<br>RTN2,<br>SLC25A4,<br>Small vasohibin-binding protein,<br>SRL,<br>SYNPO2,<br>TNNC1,<br>Tnnt2 protein,<br>Tropomyosin 1 (TPM1),<br>Tropomyosin,<br>Tubulin,<br>UDP-N-acetylglucosamine,<br>Uridine 5-monophosphate.	ALDH2<br>Azelaic Acid,<br>ß-actin,<br>ß-enolase,<br>CK,<br>ENOA,	HBA,<br>HBB,<br>Hypoxanthine,<br>LDH,<br>Nootkatone,<br>SBP2.	ACTB,<br>Adenine,<br>ALBU,<br>ASPN,<br>B2MG,<br>CAPSO,<br>CLC11,<br>CO3,<br>CO3A1,<br>CO9,<br>COBA2,<br>Collagen Type I peptides,<br>Creatine,<br>Creatinine,<br>CSPG2,<br>Cytosine,<br>D-Neopterin<br>FETUA,<br>FMOD,<br>G3P,<br>Guanine,<br>H2A1H,<br>H4,<br>HBA,	Hypoxanthine,<br>IGL1,<br>KNG1,<br>LPC(17:0)+HCOO<br>LPC(18:0)+HCOO<br>LPC(19:0)+HCOO<br>MGP,<br>MIME,<br>NUCB1,<br>PI(18:0_20:4)-H<br>PC(16:1e_20:4)+HCOO<br>PCOC1,<br>PGBM,<br>PGS2,<br>Purines,<br>Pyrimidines,<br>RCN3,<br>Taurine,<br>Thymine,<br>TTHY,<br>Uracil,<br>VIME,<br>Xanthine.
**Up-regulation pattern**	↑	1-Methylnicotinamide<br>2-Aminoethanol,<br>2-Aminoethanole,<br>3-Aminoisobutyric acid,<br>Alanine,<br>Aldehyde dehydrogenase,<br>Alpha B-crystallin,	Methionine,<br>N6-acetyl-l-lysine,<br>N-acetylneuraminate,<br>N-formylglycine,<br>Nicotinamide adenine dinucleotide hydrate	1-Methylnicotinamide,<br>3-Phenyllactic Acid,<br>Acetophenone,<br>Arginine,<br>Aspartate,<br>Choline Phosphate,<br>Histidine,	N-Acetyl-L-Phenylalanine,<br>N-acetylneuraminate,<br>N-Acetylvaline,<br>oleic acid,<br>palmitic acid,<br>Phenylalanine,<br>Putresceine,	12-Aminododecanoic acid,<br>Acetamide,<br>ANT3,<br>CHAD,	Ethyl palmitoleate,<br>N,N–Diethylethanolamine,<br>Palmitoyl ethanolamide,<br>Sedanolide.

		Arabinose,<br>Arabitol,<br>Arginine,<br>Asparagine<br>Aspartate,<br>Beta-alanine<br>Choline Phosphate,<br>Citrulline,<br>COX7B,<br>Creatinine,<br>Cysteine<br>D-alanyl-d-alanine,<br>Glucarate,<br>Gluconic acid<br>Glucosamine,<br>Glutamate<br>Glutathione S-transferase Mu2,<br>Glutathione synthetase Mu 2,<br>Glycerol phosphate,<br>Glycerol,<br>Glycerol-2-phosphate,<br>Histidine,<br>Hydroxybutyrate,<br>Hypoxanthine,<br>Hypoxanthine,<br>Indole,<br>Inositol<br>Isocitric acid,<br>Isoleucine,<br>Lactic acid,<br>Leucine,<br>Linoleic acid,<br>Lysine,<br>Malic acid,<br>Mannitol,<br>MAOB,<br>Medo-erythritol,<br>dehydrogenase flavoprotein 1,<br>N-methylethanolamine,<br>Galactosamine,<br>Octadecadienoic acid,<br>Oleic acid,<br>Ornithine<br>Palmitic acid,<br>Pantothenate,<br>Pentitol,<br>Phenylalanine,<br>Phosphate,<br>Polyubiquitin Fr. 1-73,<br>Proline,<br>Putresceine,<br>Pyroglutamate,<br>Pyroglutamic acid,<br>Rhamnose,<br>Ribitol,<br>Ribose,<br>S-benzyl-L-cysteine,<br>SERBP1,<br>Serine,<br>Serum albumin precursor,<br>Skatole,<br>SOD2,<br>Stearic acid,<br>Stress-70 protein,<br>Threitol,<br>Threonine,<br>Thymosin ß4,<br>Transferrin,<br>Tryptophane,<br>Tyrosine,<br>Uracil,<br>Valine,<br>Vimentin Fr. 443-465,<br>Xanthine,<br>Xylitol,<br>Xylose.	Indole,<br>Indole-3-Lactic Acid,<br>Leucine,<br>Linoleic acid<br>Lysine,<br>Methionine,<br>N-Acetyl-DL-tryphtophan,<br>N-Acetylhistamine,<br>Skatole,<br>Stearic acid,<br>Threonine,<br>Tryptophane,<br>Tyrosine,<br>Uracil,<br>Valine,<br>Xanthine.	

**Up-regulation followed by down-regulation**	↑↓	Aldolase A,<br>Beta-nicotinamide,<br>C-ketoacyl-CoA thiolase,<br>Creatine kinase,<br>Cytidine,<br>D-ribonucleotide,	Glutathione S-transferase alpha,<br>Reduced nicotinamide adenine dinucleotide (NADH).		
**Down-regulation followed by up-regulation**	↓↑	Arginase-1,<br>Dehydrogenase Fe-S protein 3,<br>Hydroxyacylglutathione, hydrolase,<br>Lactate dehydrogenase B,	Malate dehydrogenase 1,<br>Nicotinamide adenine dinucleotide hydrate,<br>Nicotinamide adenine dinucleotide.		
